# Systolic Nanofabrication of Super-Resolved Photonics and Biomimetics

**DOI:** 10.3390/nano10122418

**Published:** 2020-12-03

**Authors:** Konstantina Papachristopoulou, Nikolaos A. Vainos

**Affiliations:** 1 Department of Materials Science, University of Patras, 26504 Patras, Greece; vainos@upatras.gr; 2 National Hellenic Research Foundation-TPCI, 48 Vass. Constantinou Ave., 11635 Athens, Greece

**Keywords:** nanostructure, super-resolution, nanofabrication, photonics, biomimetics, bioarchitecture, aerogel, xerogel, systolic, downsizing

## Abstract

Systolic nanofabrication is demonstrated via conformal downsizing of three-dimensional micropatterned monolithic master-casts made of extremely nanoporous aerogel and xerogel materials. The porous solid skeleton collapses by thermal treatment, generating miniaturized replicas, which preserve the original stereometric forms and incorporate minified nanoscale patterns. Paradigmatic holographic and biomimetic nanoarchitectures are conformally downsized by ~4×, yielding subwavelength surface features of less than ~150 nm. The operations demonstrate the super-resolution capabilities of this alternative concept and its potential evolution to an innovative nanotechnology of the future.

## 1. Introduction

Miniaturization from the microscale towards the nanoscale is a universal technological goal. Quantum phenomena for computing, communications, and sensing require materials manipulation at the molecular and atomic level. Nanoelectronics, nanophotonics, metasurfaces, and flat optics [1] present great fundamental interest and application potential but require new super-resolution fabrication methods. Fundamental and technical impediments, however, restrict miniaturization to the planar, two-dimensional (2D) regime. On the other hand, arbitrary designs and bespoke three-dimensional (3D) device concepts are defining the new frontiers of challenging research and applications [2]. Nanofabrication is fundamentally limited by the resolution of lithographic and direct patterning methods, nominally set at half the wavelength (λ/2) of light, electrons, or ion beams used. To surpass the diffraction limit, new super-resolution techniques using advanced materials and alternative 3D nanofabrication methods thus become an unquestionable necessity.

In a different technological field, nanoporous silica aerogel and xerogel materials [3,4] have been developed for numerous and diverse applications [5]. The extreme porosity of these materials yields remarkable properties, which are revisited here in the framework of miniaturization. In our recent work [6], we patterned aerogel monoliths by direct laser writing and demonstrated that thermal sintering leads to conformal isotropic downsizing of the objects and their microstructures. In addition to aerogel micro-structuring by ArF (193 nm), Ti-sapphire (800 nm) [6], and CO_2_ (10.6 μm) laser radiation [7,8], the capabilities of molding complex monolithic aerogel sculptures [9] and optical gratings [10] have also been demonstrated.

Dimensional reduction of objects has been reported using thermo-shrinkable polymers [11], including laser processed [12], and gold-coated polystyrene [13], as well as processed photopolymers [14]. Other approaches based on hydrogel dehydration [15,16,17], some combined with complex photochemical processing [18], produced remarkable 3D microstructures. The nature of materials used and, in some cases, the observed anisotropic downsizing restrict the fabrication quality and the generic application of these methods. Thermal sintering of 3D-printed objects using eco-friendly metallic-ink and predesigned porosity demonstrated about 10% size reduction with reproducible dimensional isotropy [19]. Pyrolysis of 3D polymer micro-lattices made by ultrafast laser-writing yielded glassy carbon nanostructures with 200-nm resolution approaching 3 GPa mechanical strength [20]. In a similar approach, pyrolysis of organic–inorganic hybrid objects produced by ultrafast laser polymerization led to two-fold size reduction and 3D microstructures with sub-100-nm features [21].

In the present study, we integrated molding, nano-replication, and thermal processing using aerogels and xerogels to demonstrate, for the first time to our knowledge, a novel *systolic miniaturization concept* [22]. According to this method, arbitrarily complex 3D structures are formed by conformal downsizing of 3D solid master-objects. This approach surpasses the resolution of the lithographic or direct patterning methods used to form the original masters, thus effectively achieving super-resolution nanofabrication. In addition, the use of synthetic oxides offers unique advantages, such as chemical stability and inertness, mechanical strength, and optical quality, as well as the inherent potential for compositional tuning and doping. The paper presents this *systolic nanotechnology* using paradigms of bespoke design photonics and natural bioarchitectures.

## 2. Materials and Methods

Central to our method is the formation and processing of gels comprising silica skeletal networks. Xerogels and aerogels are air-filled extremely nanoporous solids, respectively, produced by natural or hypercritical drying of wet gels. They have unique physical properties and constitute an intriguing class of materials.

Silica gel formation was performed by hydrolysis of alkoxysilane silicon derivatives, used as standard precursors, namely tetraethoxysilane (TEOS) and tetramethoxysilane (TMOS). The synthetic part of our work addressed the degree and uniformity of the nanoporosity. Parameterization of the Si:H_2_O ratio and the acidic or basic catalytic environment enabled control of the network matrix porosity [23] and nanoparticle size [24]. In acid catalytic environments, entangled linear or randomly branched chains are usually formed, whereas, in basic environments, the network of uniform particles yields a large pore volume. Gelation time is influenced by the catalyzer and the water content, with low pH gels requiring longer time for condensation. In our work, we applied basic catalysis using ammonium fluoride (NH_4_F) [25]. The TMOS:MeOH:H_2_O:NH_4_F molar ratio of 1:12:4:3.5 × 10^−3^ M was found to yield acceptable results and further optimization is currently under way.

Sol gel synthesis [26] commenced by mixing TMOS and methanol at a molar ratio 1:12 M. In the second step, a solution of H_2_O and NH_4_F at molar ratio 4:3.5 × 10^−3^ M was added to the silica sol. The produced gel was cast in metal or polymer vessels and was left to condensate. The time for gelation was 10–20 min, in the temperature range from 60 to 20 °C, respectively.

Aging is a subsequent process that affects the structure and properties of the gel, strengthens the material, and improves its porosity [27]. We have seen that, to produce strong monolithic materials, very long aging is required. When aging is performed in the mother solution, neck growth by reprecipitation of dissolved silica occurs, with the small, dissolved particles precipitating onto larger ones. This aging process influences the microstructure of NH_4_F-catalyzed gels [28] by reorganizing the network to yield a unimodal pore size distribution.

3D solid objects were molded and patterned by replicating photonic and bioarchitectural master surfaces. Standard materials, such as polydimethylsiloxane PDMS SYLGARD™ 184 (Dow Corning GmbH, Wiesbaden, Germany) and UV-curable resins ORMOSTAMP^®^ (micro resist technology GmbH, Berlin, Germany) were used, with further intermediate steps applied to preserve pattern fidelity. Positive and negative templates were used for transferring the original surface relief patterns in aerogel and xerogel solids. The fabrication of nano-sculptured replicas of natural specimens required adaptation of alternative soft-lithography methods [29].

Materials synthesis and gel-drying are critical determinants of the final solid properties. Aerogel monoliths were produced by high-temperature hypercritical drying of alcogels using a 75 cm^3^ autoclave reactor (Parr Instruments 4740), a tubular furnace, and a gas system with electronic process control. The hypercritical drying protocol reached the temperature of 240 °C and 8 MPa pressure. Extraction of solvent by depressurization was performed above the critical point of the methanol solution at very slow rates (~10 Pa s^−1^). Under these conditions, the surface tension on the pore walls is diminished, leaving behind an intact monolithic and optically transparent nanoporous silica solid. A maximum of ~25% shrinkage was attained upon drying, depending on the synthesis and drying conditions. In all cases, the object shape was conformally preserved. Porosity was found to be in the range of 90–98%, yielding very low mass density, typically in the respective range of 0.25–0.05 g cm^−3^.

Xerogels are denser materials formed by natural drying of wet gels at room (or slightly elevated) temperatures, usually under atmospheric pressure. They have lower porosity, typically in the range of 10–70% and minimum density of ~0.7 g cm^−3^. In our work we applied drying at 60 °C under atmospheric pressure. Microscopically, when the liquid evacuates the gel body, capillary forces apply non-uniform stress on the pore walls. As a result, the network collapses into the bulk volume formerly occupied by dispersion liquid. In effect, this is the first stage of isotropic downsizing. We note that such a shrinkage is absent in aerogel formation, owing to the hypercritical conditions which null the capillary surface tension. Post-treatment at relatively low temperatures fully removes the adsorbed solvents. Further thermal processing was performed using programmable high-temperature ovens Nabertherm LTH04/16, (Nabertherm, GmbH, Lilienthal, Germany) and Thermansys BW10–1200 (Thermansys, Thessaloniki, Greece). The specific process stages are outlined in the following section for presentation clarity.

Various materials characterization methods were applied, including optical microscopy, Scanning Electron Microscopy (SEM) Zeiss EVO MA10 (Zeiss, Jena, Germany) and JEOL FESEM 7000F (JEOL Ltd., Tokyo, Japan), Atomic Force Microcope (AFM) Bruker Multi Mode employing the Nanoscope IIIa controller (Bruker, Santa Barbara, CA, USA), UV-Vis spectrometry Perkin Elmer Lambda 35 (Perkin Elmer, Waltham, MA, USA) and Shimazu UV-1900 (Shimazu Corporation, Kyoto, Japan) and FTIR spectroscopy Shimazu IRTracer-100 (Shimazu Corporation, Kyoto, Japan). The latter method monitors the presence of organics and the degree of dehydration, both being important for the subsequent sintering stages. Nitrogen porosimetry Quantachrome NOVAtouch LX^2^ (Quantachrome Instruments, Boynton Beach, FL, USA) has also been applied in selected samples to monitor the degree of porosity before and after processing. The solids produced were cast in molds and incorporated natural or designed artificial micropatterns. Optical studies were performed by use of microscopy and optics setups for photography and diffraction experiments by using a white light tungsten halogen source equipped with fiber-optic delivery cable. Diffraction efficiency of the optical grating structures was measured by use of a 10 mW He-Ne laser beam operating at 633 nm and a suitable optics set-up. This laser beam was also used for optical reconstruction of computer-generated holograms (CGH).

## 3. Results and Discussion

### 3.1. Systolic Downsizing

The synthesis of large size high-quality monolithic aerogels was a major milestone [30], which allowed the subsequent aerogel transformation into high quality glass via sintering [31,32,33] and viscous flow vitrification [34,35,36]. Great advantages are brought by the versatility of sol-gel chemistry, which enables complex glass compositions, doping, structural tuning, and environmentally friendly processing [37]. In our recent work [6], we applied laser processing [38] to form surface and in-bulk-buried microstructures in aerogels and proved that isotropic contraction of such micropatterns can be achieved upon vitrification.

Conformal systolic downsizing of sculptured and surface-tailored media was implemented in our work by molding replication and thermal treatment in distinct processing stages. Miniaturization is quantified in terms of the linear systolic factor, SF×= *L_i_*/*L_f_* = (ρ_f_/ρ_i_)^1/3^, where *L_i_* and *L_f_* are, respectively, the initial (“*i*”-before processing) and final (“*f*”-after processing) linear dimensions and ρ_f_ and ρ_i_ are the mass densities of the respective solid objects. The potential to control SF× was demonstrated and the homogeneous nature of the synthetic materials was proved to be of advantage. The aerogel processing protocol starts at the temperature of ~250 °C and proceeds to 500 °C for oxidizing the organic content. Dehydration by OH– removal produces condensation reactions that increase the network connectivity. Thereafter, diffusional sintering commences close to 900 °C. Viscous flow prevails in the final densification stage in the range ~1100 to ~1250 °C producing vitreous silica via the full collapse of the skeleton. The formed dense silica object formed is a miniaturized replica of the original nanoporous solid.

In a similar approach, systolic processing was performed in xerogels. In that case, the ambient drying process produced an initial minification that depends on the density of the original gel network. The uniformity of extraction of the solvent in this first (drying) stage is critical. In most experiments we applied drying at 60 °C, under atmospheric pressure, with a SF ~2.2× typically attained for the macroscopic (volume) object dimensions. Larger downsizing ratios were observed for surface relief features attributed to surface tension effects. For the first time to our knowledge, discrete thermal processing stages provided controlled downsizing in xerogels. A further ~1.3× reduction was achieved at 900 °C, while the final sintering stage at ~1150 °C and beyond caused an additional ~1.2× minification. The formed dense silica objects are miniaturized replicas having nanostructures which surpass the fabrication resolution of the original patterning scheme. Total linear downsizing is multiplicative, typically with linear factors SF_v_ ~3.5×–4.0× for the bulk material and SF_s_ ~5× for the surface relief. Such SF values are routinely produced in our laboratory. Figure S1 depicts relevant graphs illustrating the reduction operation in terms of actual dimensions and systolic factors. The respective measurements are presented in Section 3.2. Further optimization is in progress, targeting to the maximum dimensional reduction and replication fidelity.

Expanding the methods beyond laser patterning, we focus here on the fabrication and study of miniaturized photonic and biomimetic nanoarchitectures and present selected representative examples to illustrate the operations.

### 3.2. Photonic and Biomimetic Architectures

**Figure 1:**
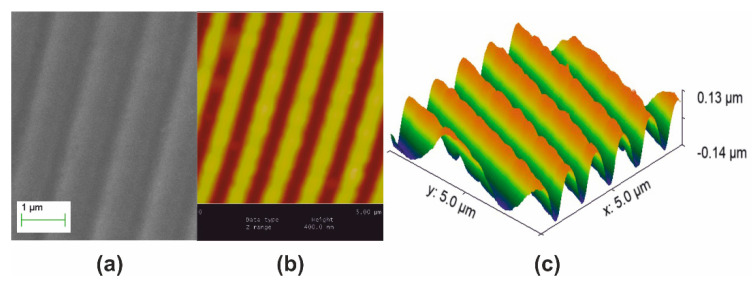
Polymeric master surface relief holographic grating used for molding aerogels and xerogels. Grating period Λ_o_ = 1 μm, groove depth ~ 200 nm: (**a**) SEM micrograph; (**b**) AFM image; and (**c**) AFM surface relief presentation.

A polymeric surface relief holographic grating master having period Λ_ο_ = 1 μm, depicted in Figure 1, was used to form monolithic aerogel holographic gratings. Figure 1a presents a SEM micrograph of the master grating. Figure 1b is its AFM image and Figure 1c is its surface relief plot using AFM data. Corrugation (groove) depth is measured at ~200 nm. 

Two fabrication modes were investigated for imprinting surface relief structures. In the first case, patterning of the exterior surface of the objects was performed. The gel was cast, and, after condensation, it was demolded and transferred to a separate vessel for aging. The second approach concerns the formation of microstructures buried in the bulk volume of the object. In this latter embedded microstructuring, both gel condensation and aging were performed in-situ, prior to demolding. Subsequently, in both cases, the aged wet-gel was transferred to the autoclave for high-temperature hypercritical drying.

**Figure 2:**
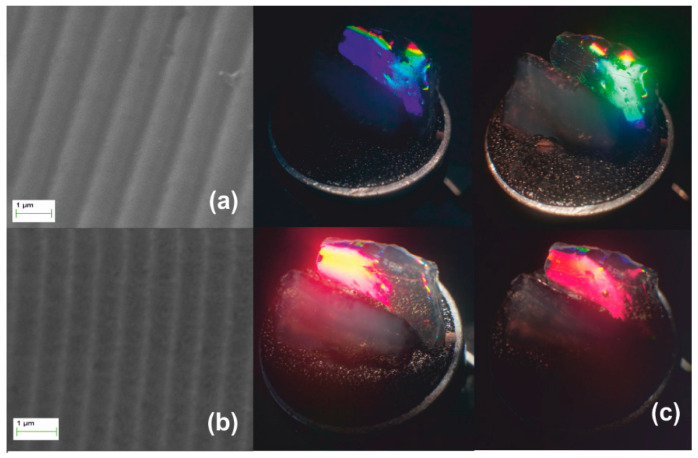
SEM micrographs of: (**a**) polymer master surface relief holographic grating of period Λ_o_ = 1 μm; and (**b**) its replica imprinted on the aerogel surface has period Λ_aero_ ~750 nm. Structural coloration by white light diffraction off the aerogel grating is observed at different angles (**c**).

Figure 2 presents an example of embedded grating replication. In Figure 2a, a SEM micrograph of the original master grating of period Λ_o_ = 1 μm is shown for comparison (see also Figure 1). As shown in Figure 2b, the aerogel monolith was sectioned into two pieces to reveal the buried grating replica of period Λ_aero_ ~750 nm. Good uniformity is maintained, even though ~25% shrinkage is recorded. We chose here a relatively dense aerogel monolith to enable efficient light diffraction for observation purposes. As shown in Figure 2c, the imprinted aerogel grating was illuminated by white light and structural colors are observed at different diffraction angles as expected.

**Figure 3:**
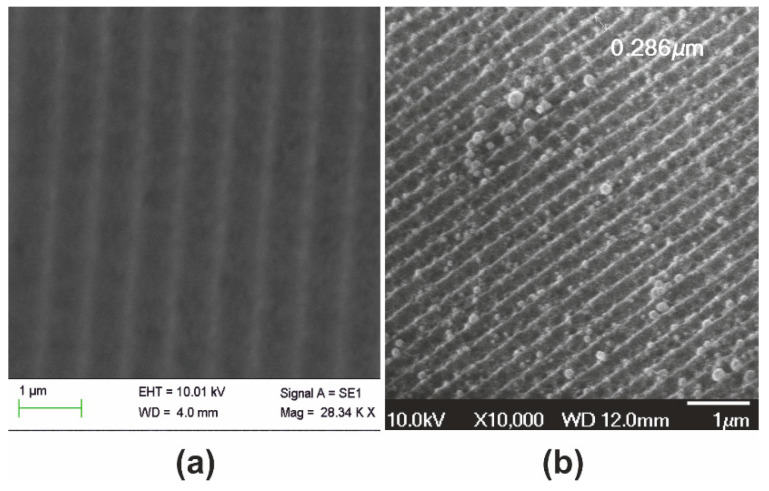
Nanostructure formation by systolic processing of aerogel grating at 900 °C: (**a**) original aerogel surface relief pattern of period Λ_aero_ ~750 nm (as in Figure 2); and (**b**) resulting surface relief pattern having grating period Λ_aero-I_ ~280 nm, downsized by a factor SF ~2.8× with respect to the original. The small droplets observed on the surface are produced by the resolidified Au used for the SEM analysis of the aerogel sample shown in (**a**).

The sample presented in Figure 2 was subsequently thermally processed in the range ~900 °C and a 2.8× dimensional reduction was observed, as presented in Figure 3.

In Figure 3a, the original aerogel surface relief grating structure having period Λ_aero_ ~750 nm is depicted for comparison to its miniaturized replica of period Λ_aero-I_ ~280 nm in Figure 3b. 

We note that the nanodroplets observed on the surface are formed by re-solidification of Au sputtered on the unprocessed aerogel sample for the SEM analysis of Figure 3a. Relevant EDX analysis of the sample in Figure 3b presented in Figure S2 verifies the presence of Au. Such effects of solidification are usually met in standard thermal processing for the fabrication of plasmonic surfaces [39].

Fabrication of xerogel structures was performed by molding and three distinct processing steps denoted as Stages I–III were subsequently applied. The first stage of natural drying of the wet gel is associated with the primary skeleton collapse. Attention was paid here to ensure uniform solvent extraction and smooth self-demolding due to dimensional mismatch.

**Figure 4:**
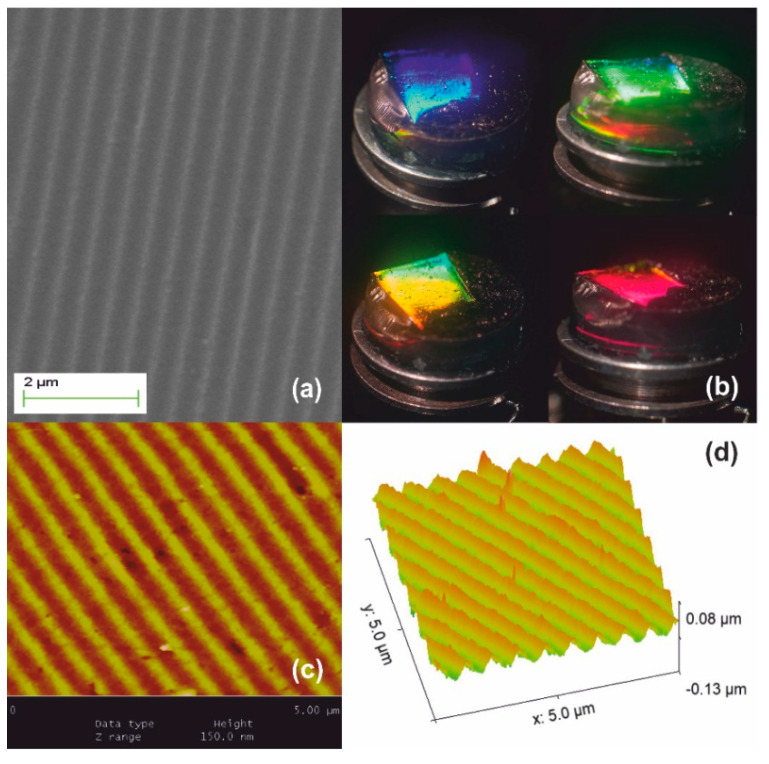
Xerogel Stage I systolic processing via natural drying: (**a**) SEM micrograph of grating with Λ_xero-I_ ~450 nm, results by SF ~2.2× contraction of the original molded gel. (**b**) The structural coloration by white light diffraction. The top-right image shows diffraction of green wavelength and undiffracted red/yellow light transmitted through the bulk. (**c**) AFM image and (**d**) AFM surface relief presentation.

Stage I systolic process yielding a monolithic xerogel replica of an optical grating (Λ_ο_ = 1 μm) is illustrated in Figure 4. The SEM micrograph of Figure 4a depicts the downsized replica of the grating imprinted in the monolith. Figure 4b presents structural colors under white light illumination. The denser material produces an enhanced grating dielectric contrast yielding diffraction efficiency of ~1.5% measured by internal incidence at 633 nm. In the top-right photograph of Figure 4b, green light is filtered out leaving the yellow-red part of the spectrum transmitted through the bulk. The produced grating recorded by AFM in Figure 4c has period Λ_xero-I_ ~450 nm, i.e., SF ~2.2× with respect to the master Λ_ο_ = 1 μm. The corrugation depth was measured in Figure 4d to less than ~60 nm, i.e., SF ~3.6× with respect to the master grating. This increased factor may be attributed to surface tension effects enhancing densification.

**Figure 5:**
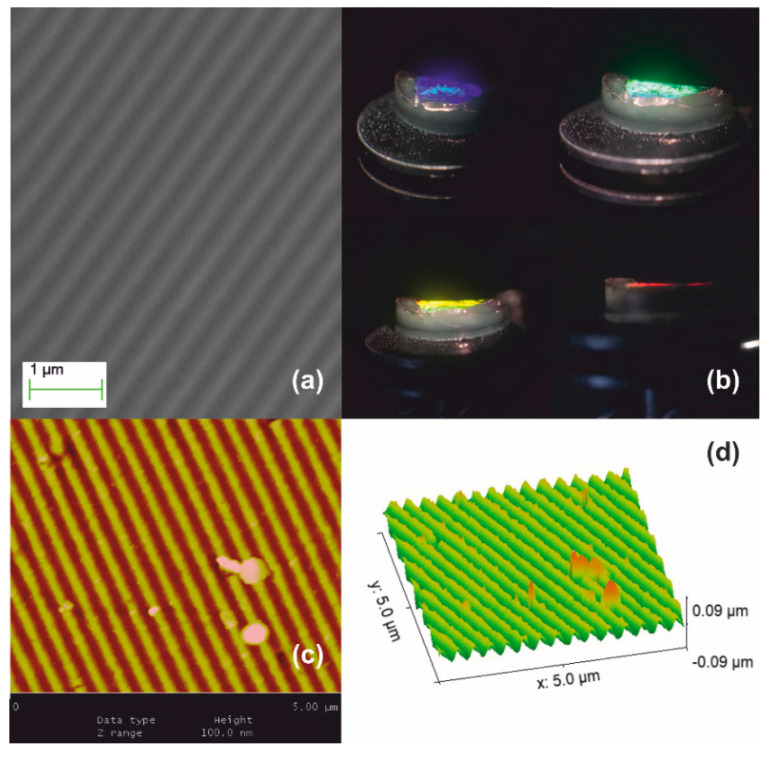
Xerogel Stage II systolic processing: (**a**) SEM micrograph of grating having Λ_xero-II_ ~ 330 nm produced by SF ~1.3× contraction of the sample depicted in Figure 4; (**b**) structural coloration by white light diffraction is visible at oblique angles of incidence; (**c**) AFM image; and (**d**) AFM surface relief presentation.

Figure 5 presents the Stage II systolic processing applied to the xerogel replica of Figure 4. In this thermal pre-sintering, we applied slow heating ramps through the 250, 500, and finally 950 °C temperature ranges. Treatment from 500 to 700 °C causes further contraction to Λ_xero-II_ ~330 nm by SF ~1.3×, as in Figure 5a. As expected, the structural coloration shifts towards the short wavelength range, as depicted in Figure 5b. Diffraction is observed at quite oblique incidence within a diminished field of view, the red spectrum becoming barely visible (lower right-hand image). AFM analysis in Figure 5c provides grating period measurement and grating (corrugation) depth of less than ~50 nm, i.e., SF ~1.2× reduction of surface relief, as shown in Figure 5d.

**Figure 6:**
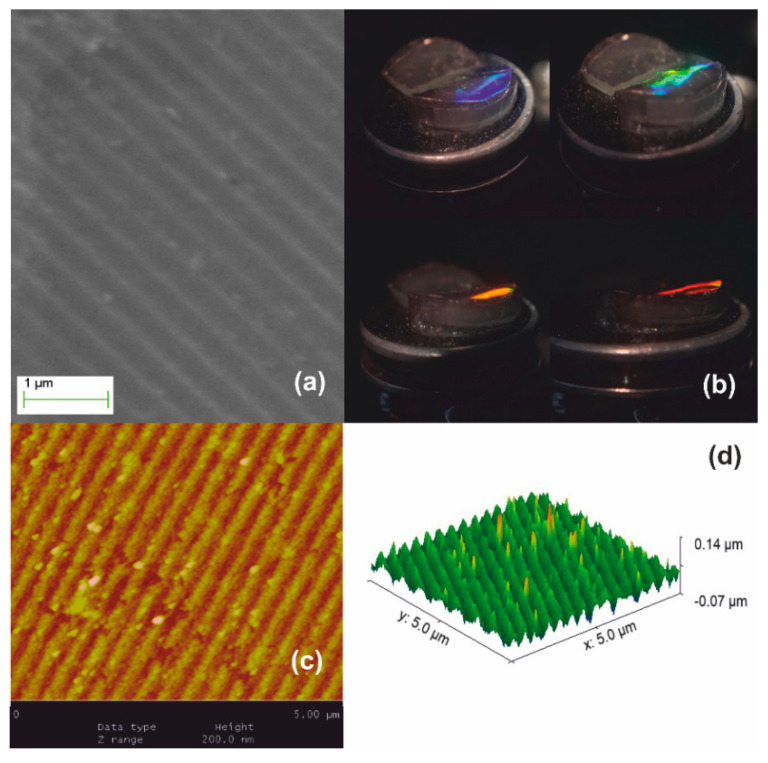
Xerogel Stage III systolic processing yields further 1.2× size reduction: (**a**) SEM image of the downsized replica having period Λ_xero-III_ ~280–300 nm; (**b**) white light diffraction produces structural colors observable by internal incidence at very oblique angles; (**c**) AFM image; and (**d**) AFM surface relief presentation.

Stage III processing is illustrated in Figure 6. The material densifies further beyond 950 °C and viscous flow fully collapses the network at ~1150 °C forming fused silica. Further dimensional reduction of SF ~1.2× is achieved producing the ultimate surface relief silica-glass grating having period Λ_xero-III_ ~280–300 nm, as measured by AFM in Figure 6c. Corrugation depth is at ~40 nm implying a ~1.2× systolic factor as well in Figure 6d. This result corroborates the global uniformity of downsizing at high temperatures. White-light diffraction was only possible by internal incidence, i.e., from solid to air and diffracted light is only observed at very oblique incidence, as depicted in Figure 6b. The field of view further decreases, and the limiting orange-red wavelength region is barely observable in a minute angular range (lower right-hand image). The diffraction efficiency is measured at ~3% by oblique internal incidence at 633 nm.

The total systolic miniaturization factor is given by the multiplication of systolic factors of each downscaling step. For the bulk linear dimensions SF_v_ ~3.6× and for the surface relief SF_s_ ~5× are estimated. An account of the observed systolic factor upon thermal treatment is given in Figure S1.

**Figure 7:**
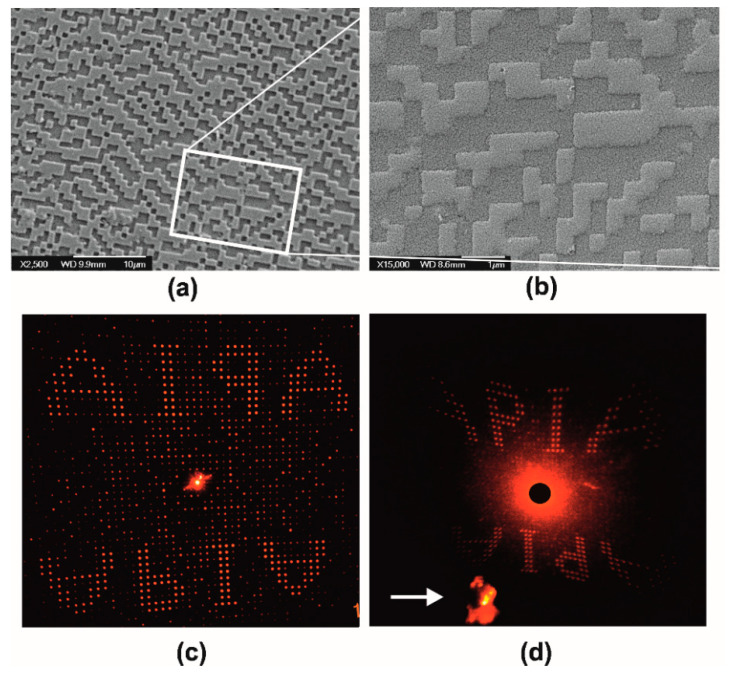
Stage I miniaturization of computer-generated surface relief hologram (CGH) in xerogel: (**a**) polymer resist master is a negative replica of the original structure on silicon; (**b**) SF ~2.5× downsized xerogel replica of the pattern in (**a**), where CGH structures were reconstructed by using a He-Ne laser beam operating at 633 nm; (**c**) reconstructed image of reflective CGH of the original silicon master; and (**d**) reconstructed image of transmission CGH xerogel replica in (**a**) indicated by a white arrow in image (**d**).

Figure 7 illustrates the systolic processing of a surface relief binary-phase computer-generated hologram (CGH). Such diffractive optical elements encode optical information and find several applications in laser beam forming, sensing, information processing, and telecom interconnects. The specific GCH was designed using simulated annealing algorithms and the pattern was transferred on a silicon wafer by e-beam lithography and reactive ion etching. This master surface was copied by soft lithography in two pattern transfer steps using PDMS and ORMOSTAMP^®^. Figure 7a depicts the negative replica of the original silicon master CGH transferred in ORMOSTAMP^®^ resin. This negative has 1 μm × 1 μm pixel size (Λ_ο_ = 2 μm) and it was used for xerogel molding. Stage I processing yields the miniaturized replica of the original CGH is downsized by SF ~2.5× at ~400 nm × 400 nm pixel size (Λ_xero-I_ = 0.8 μm), as presented Figure 7b. The exact hologram area in Figure 7a is framed for clarity. 

In Figure 7c, the optical reconstruction (reflective mode) of the master CGH using a HeNe laser is shown. Scattering angle for the first order images “APIA” is at 18°. Figure 7d presents the respective reconstruction of the miniaturized CGH in transmission mode. The actual CGH sample is marked with an arrow. The image deformation is due to the large ~50° scattering angle. Figure S3 presents the original silicon master and relevant optical reconstructions obtained in reflection and transmission.

**Figure 8:**
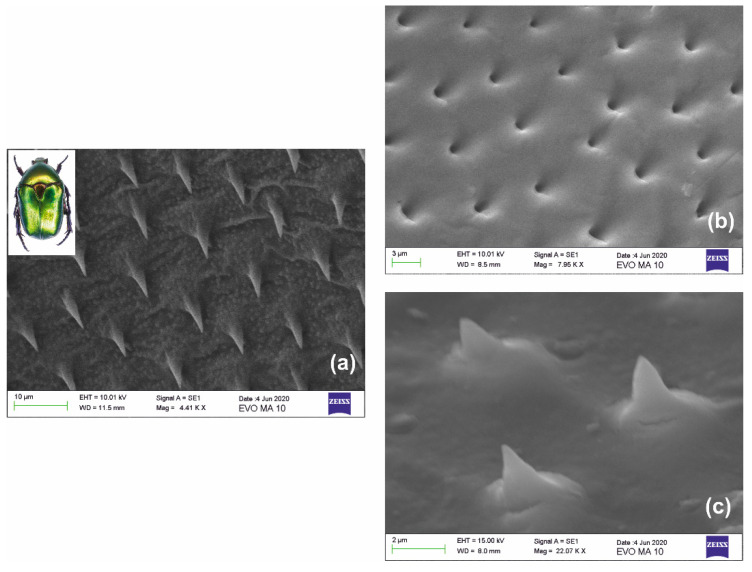
Miniaturization of a biomimetic structure: (**a**) SEM images of natural needles found in the wing shell of coleopteron protaetia cuprea phoebe (inset); (**b**) miniaturized negative replica of the structure in xerogel; and (**c**) miniaturized positive replica of the needles in xerogel. Both (**b**,**c**) have undergone SF ~4× maximum downsizing. The observed size of the apex of the conical needles is restricted by the intermediate replication stage and the electron microscope resolution limit.

Extending the above approach, we focus on the case of optoelectronic sensors with transparent nano-tips probing near-field interactions. In this context, our paradigm of biomimetic nanofabrication uses the intriguing wing-shell structures found in coleopteron *Protaetia Cuprea Phoebe* [40], a member of the *Cetoninae Scarabaeidae* family. The *“golden beetle”* depicted in the inset of Figure 8a is found in abundance in Greece and the Mediterranean, as well as in other regions of Europe and Asia.

The structure of interest is the ensemble of microneedles depicted in Figure 8a. They have a conical form of 15–20 μm height and elliptical or circular cone base of ~5–6 μm diameter and are regularly positioned ~10–15 μm apart quasi-periodically, in some areas forming a perfect hexagonal lattice. Figure 8b presents the Stage I processing of the negative xerogel replica directly casted using the natural surface. In the second approach, an intermediate step of imprinting was applied to cast a negative master in ORMOSTAMP^®^. This negative master was subsequently used to form the positive replica depicted in Figure 8c. The fabricated bioarchitectural form is a downsized replica having cone height of <~4 μm and cone base diameter of ~1.5 μm, thus implying SF ~4× miniaturization with excellent reproduction fidelity. Considering the reduction factor, the actual size of the cone apex is estimated to be less than ~30 nm, restricted by the intermediate replication process. Imaging of the cone apex is limited by the resolution of the SEM instrument.

The above results justify the great potential of this concept in terms of fabrication resolution and flexibility. First, the casting of 3D porous objects using micro- and nano-structured milled, laser written, lithographed, or natural masters is verified. Second, post-sculpturing offers a further degree of freedom and provides additional functionalities. Third, systolic processing can achieve pattern resolution beyond the capacity of the original mastering methods. The combination of this method with novel synthetic materials will place the scheme in the forefront of future 3D manufacturing nanotechnologies.

## 4. Conclusions

A novel systolic nanofabrication methodology was demonstrated based on the isotropic downsizing of micropatterned, extremely nanoporous solids. Thermal processing of silica aerogel and xerogel masters resulted in conformal size-reduction, preserving stereometry and incorporated microstructural forms. Paradigms of nanoscale functional structures demonstrated fabrication with high fidelity and super-resolution. Distinct processing stages provided stepwise miniaturization with multiplicative downsizing. Total linear systolic factors of ~4× at maximum for volume and 5× for surface relief features were attained and further optimization is currently in progress. Paradigmatic fabrication of surface relief holographic gratings and computer-generated holograms highlights the potential of subwavelength photonics and other novel functionalities. In addition, natural bioarchitectures produced unique 3D biomimetic nanostructures for potential use in photonic sensor, biomedical, and micro-hydrodynamics applications. The inherent potential for controlling the physical and chemical properties of the materials, combined with the capabilities for super-resolution fabrication in 3D space, would establish this beyond the state-of-the-art 3D-nanomanufacturing technology.

## Supplementary Materials

The following are available online at https://www.mdpi.com/2079-4991/10/12/2418/s1, Figure S1: Dimensional reduction vs. process temperature, Figure S2: Au droplet formation. Figure S3: Silicon CGH master and optical reconstructions.
