# Supplementary material for: Systolic Nanofabrication of Super-Resolved Photonics and Biomimetics
Source: Nanomaterials (Basel). 2020 Dec 3;10(12):2418. doi: 10.3390/nano10122418 (PMC7761641; doi:10.3390/nano10122418)
Supplement: Supplementary file 1 [file nanomaterials-10-02418-s001.pdf]

Supplementary Materials

Nanomaterials Manuscript No 970662:
"Systolic nanofabrication of super-resolved photonics and biomimetics"
by K. Papachristopoulou and N.A. Vainos

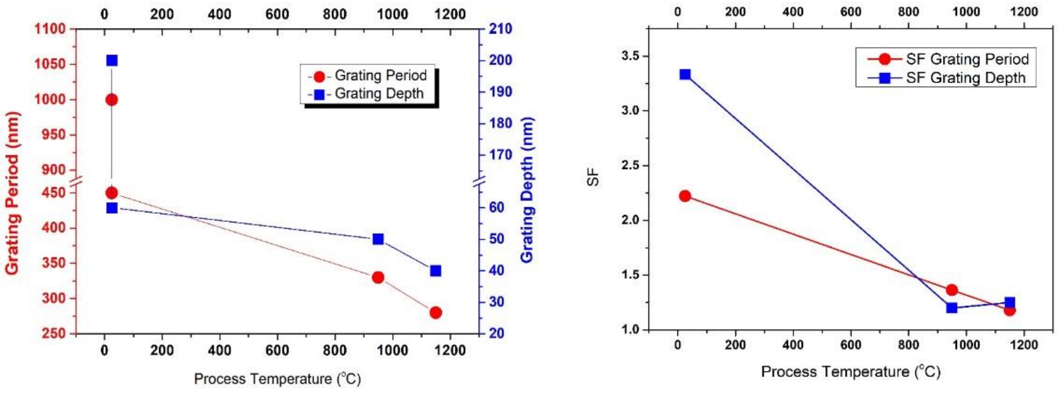

(a) (b)

Figure S1: Dimensional reduction vs.process temperature. (a) Surface relief grating period and grating depth in xerogel as a function of process temperature. **(b)** Respective Systolic Factor (SF) of grating period and depth on xerogel surface as a function of the process temperature. Overall size reduction is given by the multiplication of step systolic factors found as SF_v ~3.6 for the lateral linear dimensions (volume) and SF_s~5 for the linear surface relief.

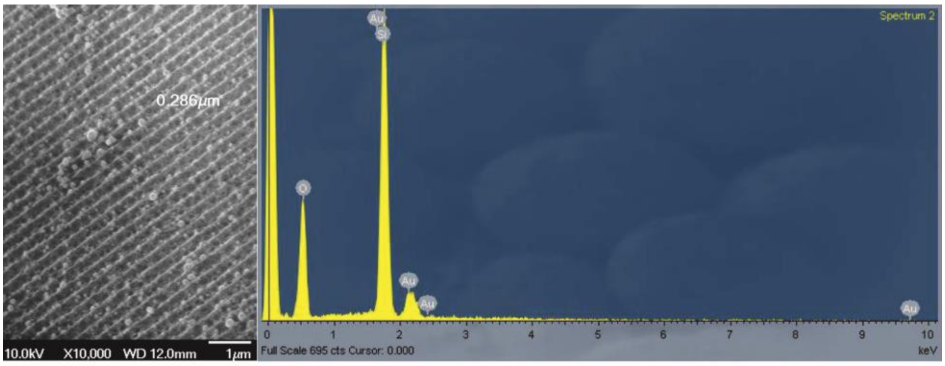

(a) (b)

Figure S2: Au droplet formation. (a) Miniaturized grating replica of period Λ_{aero-I} ~ 280 nm formed by systolic processing of cast aerogel grating by sintering at 900 °C. The small droplets observed on the surface are produced by precipitation of Au applied for the SEM analysis of the unprocessed aerogel sample **(b)** EDX analysis of the sample in (a) demonstrating the presence of Au and silica elements.

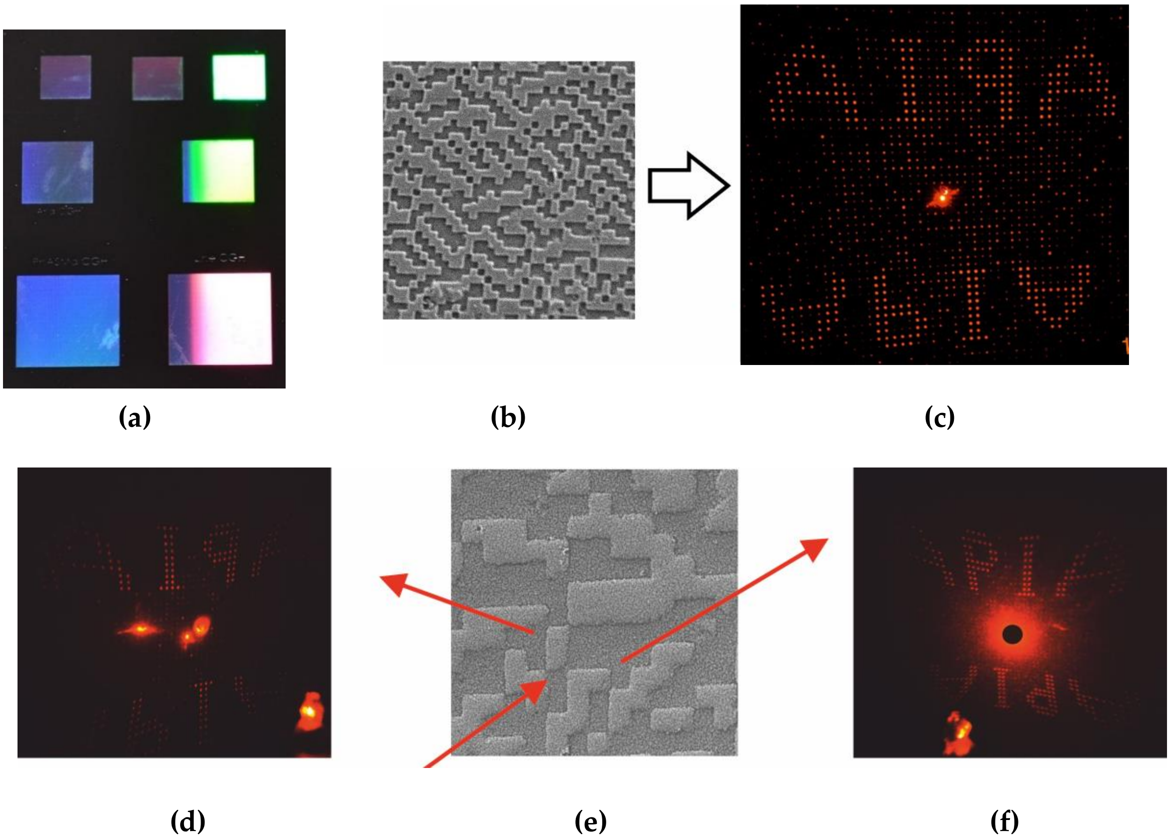

(a) (b) (c) (d) (e) (f)

Figure S3: Silicon CGH master. (a) photo of original silicon master of surface relief binary-phase computer-generated holograms (CGH) showing diffraction colors under white light illumination, (b) SEM image of surface relief CGH, (c) reflective CGH reconstruction at 633 nm, (d) reflective mode CGH reconstruction at 633 nm, (e) SEM image of surface relief CGH on xerogel and (f) transmissive mode CGH reconstruction at 633 nm.
